# Instantaneous global horizontal irradiance and clearness index data with high temporal resolution for 1016 days

**DOI:** 10.1016/j.dib.2022.108504

**Published:** 2022-08-02

**Authors:** Alberto Avila, Rafael Diez

**Affiliations:** Electronics Engineering Department, Facultad de Ingeniería, Pontificia Universidad Javeriana, Edificio 42, Cra. 7 No. 40-62, Bogotá, Colombia

**Keywords:** Solar irradiance, Photovoltaic system, Stability of the radiative regime, Irradiance variability, Solar energy, Pyranometer, Power electronics

## Abstract

Global Horizontal Irradiance was measured using a thermopile-type pyranometer during more than three years using a sample time of two seconds, with the purpose of capturing fast transient events of irradiance which are notable in tropical regions as the one where these data were collected: Bogotá, Colombia.

The date and time of each measurement were registered along with the irradiance values. In addition, other related quantities were calculated and included for each one of the measurement instants: Optical Air Mass, Zenith angle, Extraterrestrial Solar Irradiance, and Clearness Index. Daily aggregated statistics of irradiance were calculated and are provided here too.

Data points corresponding to nights were discarded. The raw data was analyzed to remove incomplete days, to guarantee that daily statistics are accurate and meaningful. After this data cleaning process, 1016 complete days remain, having a total of 21,959,912 data points.

These data are useful for studying the effect of irradiance transients over photovoltaic systems, including power electronics, batteries and electric loads; it can also be used in studies about the stability of the radiative regime or the variability of irradiance such as Avila et al. (2019) (where part of these data was effectively used) and other related works cited there.

## Specifications Table

**Table:** 

Subject	Energy
Specific subject area	Renewable Energy, Sustainability and the Environment
Type of data	Table
How the data were acquired	Global Horizontal Irradiance was measured with a thermopile-type pyranometer (Kipp & Zonen CM3) during more than three years, between 2016-08-19 and 2019-11-10. Data was recorded with a sample time of 2 s (sample rate of 0.5 Hz), using a data-logging device and software from the same manufacturer as the instrument (Kipp & Zonen SOLRAD Radiation Indicator).
Data format	Table “irradiance_data”: Raw. Table “daily_stats”: Analyzed. Useful quantities were calculated and added to the “irradiance_data” table, for each one of the data points: Zenith angle, Air Mass, Extraterrestrial Irradiance ($I_{0h}$) and Instantaneous Clearness Index ($k_{t}$). Aggregated statistics for each day were calculated and are included in the “daily_stats” table.
Description of data collection	Irradiance ($I_{s}$) was continuously measured, but due to sporadic technical difficulties a few data points were lost. Therefore, to have reliable statistics of daily totals, the days missing relevant data were completely removed from the tables; 1016 days were kept from the total that was measured. Data were considered relevant in the time range where the calculated Extraterrestrial Irradiance ($I_{0h}$) was greater than zero, that is, when sun light is expected.
Data source location	• Institution: Pontificia Universidad Javeriana • City/Town/Region: Bogotá • Country: Colombia • Latitude: 4 ° 35′ N, Longitude: 74 ° 40′ W
Data accessibility	Repository name: Mendeley Data Data identification number: DOI: 10.17632/56jcrt85dt.1 Direct URL to data: https://doi.org/10.17632/56jcrt85dt.1
Related research article	[1] A. Avila, P. R. Vizcaya, and R. Diez, “Daily irradiance test signal for photovoltaic systems by selection from long-term data,” *Renewable Energy*, vol. 131, 2019, doi:10.1016/j.renene.2018.07.071.

## Value of the Data

• The data was measured using a high sampling rate, which allowed to capture many fast transient behaviors of received irradiance, produced by atmospheric phenomena. Also, longer term phenomena are present in the data since it was taken over a period of more than three years.
• Researchers working on photovoltaic systems, testing of power electronics for energy conversion, or the modelling of the solar resource can find these data useful.
• From these data, the transient behavior of irradiance can be characterized and its impact in photovoltaic systems can be assessed, including power electronics, battery systems, electric loads, etc.
• By Integration of the data, it is possible to obtain energy totals, needed in the sizing of solar energy systems.
• Also, it can be used to create statistical models of irradiance levels, or synthesis of new irradiance signals; as done, for example, in [2].

## 1 Data Description

Solar irradiance at the surface of the earth is heavily influenced by atmospheric phenomena like clouds, which can produce fast and considerable variations in the received irradiance [3], this is more notable in tropical regions. Therefore, to be able to account for these sudden changes, data measured with a high sample rate (i.e., low sample time) is necessary [4].

These data are provided here on the table “irradiance_data”, along with other useful quantities. Daily aggregated statistics are calculated on the table “daily_stats”. Both tables can be found on [5].

**Fig. 1:**
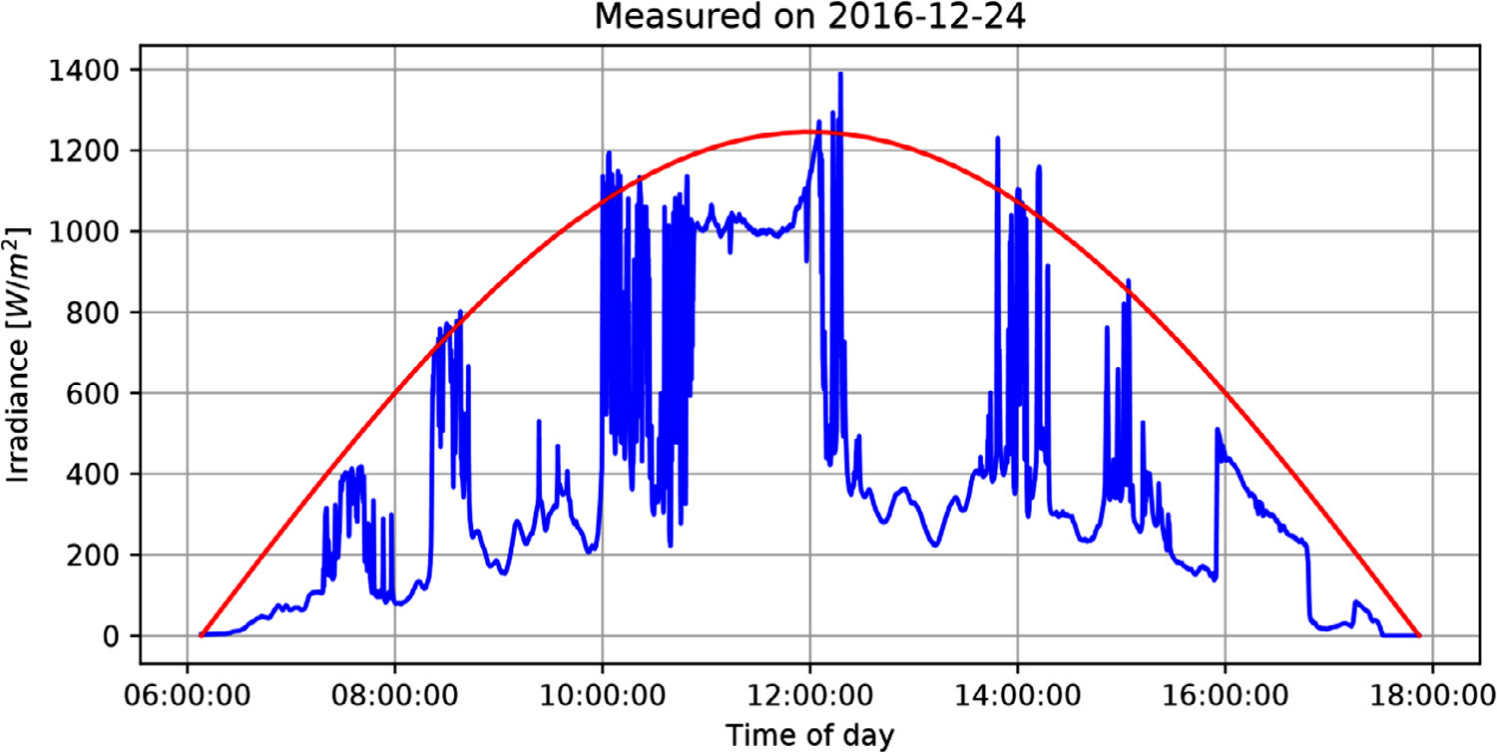
Measured irradiance (blue) and calculated extraterrestrial irradiance (red) for 2016-12-24.

An example of the data available for one single day (of the 1016 available) including only Global Horizontal Irradiance (blue) and Extraterrestrial Solar Irradiance (red) is shown in Fig. 1.

**Table:** Table: “irradiance_data”

*Records:* 21,959,912 irradiance measurements
*Columns with example values inside single quotes:*
date_and_time: '2016-12-24 12:00:00′
irradiance: '1140.5′ in *W/m*^2^
meas_date: '2016-12-24′
meas_time: '12:00:00′
am: '1.13205′ Air mass
zenith_rad: '0.48951′ Zenith angle in radians
h0: '1245.9′ Extraterrestrial Solar Irradiance in *W/m*^2^
kt: '0.915401′ Clearness index

**Table:** Table: “daily_stats”

Records: 1016 days
*Columns with example values inside single quotes:*
meas_date: '2016-12-24′
i_count: '21121′ irradiance points for the day
i_min: '0.0′ daily minimum irradiance
i_max: '1390.0′ daily maximum irradiance
i_avg: '377.623′ daily average irradiance
i_std: '321.279′ daily standard deviation of the population
dawn: '06:08:00′ first measured time
dusk: '17:52:00′ last measured time

## 2 Experimental Design, Materials and Methods

**Fig. 2:**
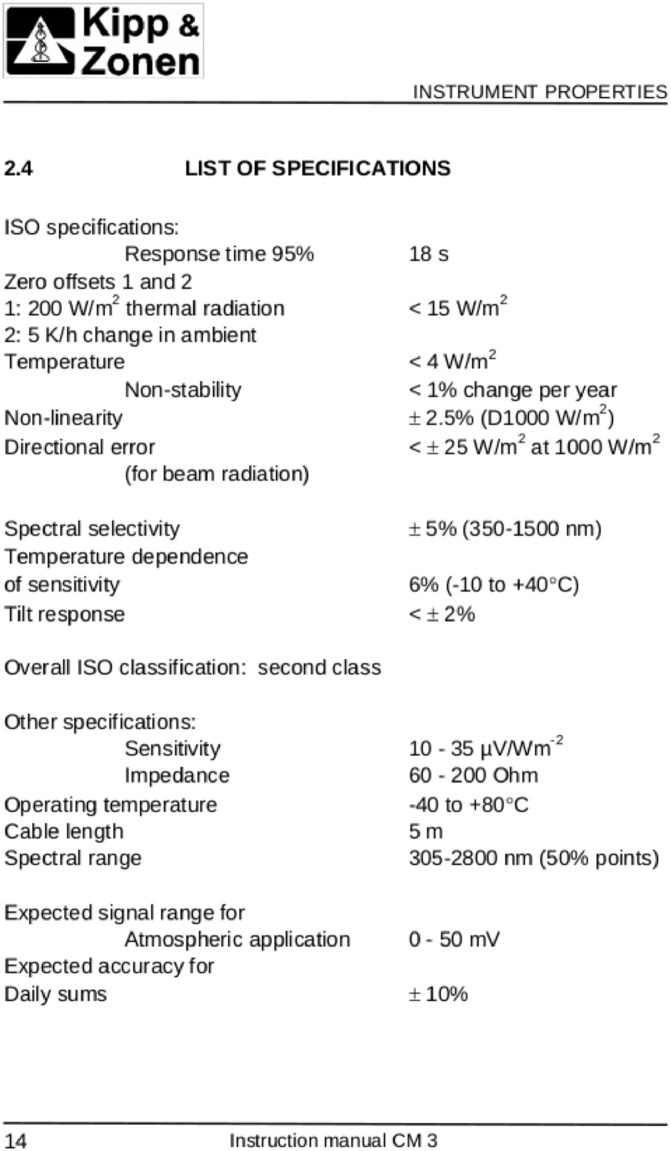
Pyranometer specifications.

The thermopile-type pyranometer was used to measure Global Horizontal Irradiance between the dates 2016-08-19 and 2019-11-10. Specifications of this instrument are shown in Fig. 2.To capture fast transient phenomena, a sampling time of 2 *s* was used, which is considered appropriate for instantaneous irradiance measurements [4].

Raw data registered by the recording software, was transformed to a standard format much more accessible for data analysis tools. For each data point three values were recorded: date, time, and Global Horizontal Irradiance ($I_{s}$).

The Zenith angle, Air Mass, and Extraterrestrial Solar Irradiance ($I_{0h}$) were calculated for each data point, based on the geographical coordinates, date and time of the measurement, using the formulas available at Sections 3.6 and 3.11 of [6]. This was also used to calculate the Instantaneous Clearness Index as defined in [3]: *k_t_ = I_S_/I_0h_*.

Data corresponding to non-positive values of $I_{0h}$ were discarded, since this corresponds to negligible values of measured $I_{s}$, below the accuracy of the pyranometer.

Days with any missing data were completely removed since this would create defective irradiance signals or daily statistics. Afterwards, aggregated statistics were calculated for each one of the 1016 days, as given in table “**daily_stats**”.

## Ethics Statements

The presented data involved none of the following: human subjects, animal experiments, or data collected from social media platforms.

## CRediT authorship contribution statement

**Alberto Avila:** Software, Data curation, Writing – review & editing. **Rafael Diez:** Validation, Writing – review & editing.

## Declaration of Competing Interest

The authors declare that they have no known competing financial interests or personal relationships that could have appeared to influence the work reported in this paper.

The authors declare the following financial interests/personal relationships which may be considered as potential competing interests: There are not any other interests to declare.
